# Genome-Wide Characterization of the WIP Transcription Factor Gene Family in Soybean and Physiological Responses to Salt Stress

**DOI:** 10.3390/genes17080968

**Published:** 2026-08-18

**Authors:** Tianjiao Gao, Shuping Yan, Sobhi F. Lamlom, Huilong Hong, Tiantian Huang, Guoqing Li, Narentuya Chen, Chunlei Zhang, Honglei Ren, Qiang Qiu, Lichun Huang

**Affiliations:** 1 Hinggan League Institute of Agricultural and Husbandry Sciences (Inner Mongolia Innovation Center of Biological Breeding Technology), Ulan Hot City 137400, China; gaotianjiao2000@163.com (T.G.);; 2 Soybean Research Institute, Heilongjiang Academy of Agriculture Sciences, Harbin 150086, China; 3 Resource Utilization and Plant Protection, College of Modern Agriculture and Ecological Environment, Heilongjiang University, Harbin 150080, China; 4 Plant Production Department, Faculty of Agriculture Saba Basha, Alexandria University, Alexandria 21531, Egypt; 5 National Key Facility for Gene Resources and Genetic Improvement, Key Laboratory of Crop Germplasm Utilization, Ministry of Agriculture, Institute of Crop Science, Chinese Academy of Agricultural Sciences, Beijing 100081, China; 6 Soybean Research Institute, Jilin Academy of Agricultural Sciences (Northeast Agricultural Research Center of China), Changchun 130033, China

**Keywords:** *Glycine max*, WIP transcription factor, C2H2 zinc finger, gene family evolution, salt stress, gene expression profiling, cis-acting elements

## Abstract

Background/Objectives: Soybean (*Glycine max*) productivity is increasingly constrained by soil salinity. WIP transcription factors, a subfamily of C2H2-type zinc finger proteins, regulate cell division, differentiation, and tissue patterning in several plant species, but this gene family had not previously been systematically characterized in soybean or any other major legume crop. This study aimed to identify and characterize the *GmWIP* gene family genome-wide and evaluate its potential involvement in the soybean salt-stress response. Methods: Genome-wide identification of *GmWIP* genes was performed using sequence similarity and domain-based searches against the Wm82.gnm4.ann1 reference genome, followed by characterization of physicochemical properties, chromosomal distribution, phylogenetic relationships, gene duplication, conserved motifs, gene structure, and promoter cis-acting elements. Tissue-specific expression was examined using transcriptome data, and *GmWIP* responses to salt stress were profiled by RT-qPCR in roots, stems, and leaves of a salt-tolerant cultivar (HN531) and a salt-sensitive cultivar (HN563), alongside physiological measurements of oxidative stress and osmotic adjustment. Results: Thirty *GmWIP* genes were identified, with molecular weights from 26.90 to 57.52 kDa, distributed unevenly across 15 soybean chromosomes, with chromosomes 11, 12, and 13 forming a major hotspot (53.3% of the family). Duplication analysis detected 54 reconciled segmental duplicate gene pairs, all exhibiting Ka/Ks values < 1 (ranging from 0.0351 to 0.4471; mean 0.214), consistent with purifying selection acting on this gene set. GmWIP promoters were enriched for ABRE, MBS, and MeJA cis-acting elements. RT-qPCR showed genotype- and tissue-dependent differential expression under salt stress (e.g., up to 14.9-fold induction of *GmWIP22* in HN531 stems), paralleled by superior proline accumulation (+45%), soluble sugars, and CAT activity (+38%) alongside reduced MDA accumulation in the tolerant cultivar. Conclusions: The *GmWIP* gene family has expanded substantially in soybean relative to previously characterized species and shows genotype-dependent transcriptional responses to salt stress, suggesting that specific *GmWIP* members are candidate regulators of salt tolerance and warrant further functional investigation.

## 1. Introduction

Transcription factors containing C2H2-type zinc finger domains constitute one of the largest and most functionally diverse families of DNA-binding regulatory proteins in plants, with individual members contributing to processes ranging from meristem maintenance and organ patterning to responses against abiotic and biotic stress [1,2]. Within this broader superfamily, the WIP subfamily is distinguished by a characteristic arrangement of four zinc finger domains and is classified within the A1d subgroup of C2H2 zinc finger proteins, a subgroup that is structurally distinct from the more extensively studied QQ-type and single-finger C2H2 proteins involved in general stress signaling [3,4]. WIP genes have been identified in every sequenced plant genome examined to date, suggesting that this subfamily arose early in land plant evolution and has since been retained across diverse lineages [5]. Despite this apparent conservation, functional characterization of WIP genes has so far been reported in only a limited number of species, and the extent to which WIP-mediated regulatory functions are conserved, diversified, or expanded across major crop lineages remains incompletely understood.

In *Arabidopsis thaliana*, the WIP family comprises six members, WIP1 through WIP6, of which single-mutant phenotypes have been described for three (wip1, wip2, and wip6) [6]. The founding member, WIP1/TRANSPARENT TESTA 1 (WIP1/TT1), is expressed in the seed coat endothelium during seed development, and its loss of function produces yellow seeds that lack proanthocyanidins, in contrast to the brown seed coat characteristic of wild-type plants [6]. WIP6, also known as DEFECTIVELY ORGANIZED TRIBUTARIES 5 (DOT5), has been associated with altered leaf venation patterning, delayed leaf and root development, and occasional changes in phyllotaxy [7]. WIP2 is the most extensively characterized member of the family and is required for transmitting tract development in the gynoecium; its loss of function gives rise to the no transmitting tract (ntt) mutant [8]. WIP2 overexpression increases replum cell number and causes fruit indehiscence [9,10], and WIP2 physically interacts with the MADS-box protein SEEDSTICK (STK), with the stk ntt double mutant displaying septum fusion defects not observed in either single mutant [11]. These findings collectively indicate that WIP transcription factors in Arabidopsis participate in a range of developmental processes spanning seed coat pigmentation, vegetative organ patterning, and reproductive tissue differentiation.

Functional roles for WIP genes outside Arabidopsis have also been documented, though in a limited number of species. In melon (*Cucumis melo*), *CmWIP1* expression has been linked to carpel abortion and the promotion of male flower development, and epigenetic modification of the *CmWIP1* promoter is associated with the occurrence of pistillate or hermaphroditic flowers, implicating this gene in the regulation of sex determination [12]. In gerbera (*Gerbera hybrida*), a WIP-family zinc finger protein has been shown to suppress cell expansion by mediating crosstalk among gibberellin, abscisic acid, and auxin signaling pathways [4]. Together with the Arabidopsis studies, these reports suggest that WIP transcription factors have been co-opted for distinct, lineage-specific developmental functions in different plant species, even as the core protein architecture of the family remains conserved [13].

Despite this evidence for functional diversification, systematic genome-wide characterization of the WIP gene family has not, to our knowledge, been reported in any major legume crop. Soybean is among the most economically important crops worldwide, providing a primary source of dietary protein and vegetable oil for both human consumption and animal feed [14,15]. Soybean production is substantially constrained by soil salinity across many cultivation regions, and this constraint is expected to intensify as irrigation practices, groundwater salinization, and the expansion of agriculture onto marginal land increase the proportion of saline-affected soils under cultivation [16,17]. At the physiological level, salt stress disrupts cellular ion homeostasis, imposes osmotic stress that limits water uptake, and promotes the accumulation of reactive oxygen species, which in turn damages membrane lipids, proteins, and nucleic acids unless countered by osmotic adjustment and antioxidant defense mechanisms [18,19]. The degree to which a given genotype can maintain ion balance, osmotic adjustment, and redox homeostasis under saline conditions is a major determinant of its overall salt tolerance, and genotypic differences in these traits are frequently exploited as selection criteria in salt-tolerance breeding programs [20]. These physiological responses are themselves under transcriptional control, and several transcription factor families, including members of the AP2/ERF, bZIP, MYB, and NAC families, have been implicated in coordinating salt-stress responses in soybean and other crop species [21,22]. Whether members of the WIP family, which have not previously been examined in this context, contribute to salt-stress signaling or tolerance in soybean has not been addressed.

In this study, we conducted a genome-wide identification and characterization of the WIP transcription factor gene family in soybean. Using sequence similarity and domain-based searches against the soybean genome, we identified the complete complement of *GmWIP* genes and characterized their physicochemical properties, chromosomal distribution, phylogenetic relationships relative to Arabidopsis WIP proteins, gene duplication history, conserved motif composition, exon-intron structure, and promoter cis-acting elements. To evaluate the potential involvement of *GmWIP* genes in growth, development, and stress responses, we further examined their expression patterns across eleven soybean tissues using publicly available transcriptome data. Finally, to directly assess the responsiveness of this gene family to salt stress, we profiled *GmWIP* transcript abundance by RT-qPCR in the roots, stems, and leaves of a salt-tolerant cultivar (HN531) and a salt-sensitive cultivar (HN563) under control and salt-stress conditions, and we complemented these expression data with physiological measurements of oxidative damage, osmotic adjustment, and antioxidant enzyme activity in the same tissues. Together, these analyses provide a genomic and expression resource for the soybean WIP family, offer insight into its evolutionary history relative to Arabidopsis, and identify candidate *GmWIP* genes for future functional investigation in the context of soybean salt tolerance and, potentially, for use as molecular markers in breeding programs targeting improved salt tolerance.

## 2. Materials and Methods

### 2.1. Genome-Wide Identification of WIP Family Genes

Six Arabidopsis WIP protein sequences were downloaded from TAIR (https://www.arabidopsis.org/, accessed on 22 April 2026) and used as BLASTP queries against the soybean genome with an E-value cutoff of 1 × 10^−20^ and a score above 100 [3]. HMM profiles for the diagnostic C2H2 zinc finger domain (Pfam: PF00096) were obtained from Pfam (https://pfam.xfam.org/) and searched against all soybean protein sequences using the soybean reference genome *G.max* Wm82.gnm4.ann1 (Phytozome v13) via HMMER ($E<1\times 10^{-20}$). Candidate proteins from both approaches were filtered for the presence of conserved domains using Pfam, SMART, and CDD (https://www.ncbi.nlm.nih.gov/cdd/, accessed on 22 April 2026) via NCBI. Retained proteins were verified by searching against the Arabidopsis genome to exclude non-WIP family members. Subcellular localization was predicted using WoLF PSORT and CELLO v2.5. The 30 identified *GmWIP* genes were renamed according to their chromosomal positions (*GmWIP1* through *GmWIP30*). Physicochemical parameters, including molecular weight, theoretical isoelectric point, instability index, aliphatic index, and GRAVY, were computed via ExPASy ProtParam.

### 2.2. Multiple Sequence Alignment and Phylogenetic Analysis

Amino acid sequences of six AtWIP and 30 GmWIP proteins were aligned using ClustalW in MEGA12 [23]. Phylogenetic trees were constructed using the Maximum Likelihood (ML) method based on the JTT matrix-based model with gamma-distributed substitution rates (JTT+G), supported by 1000 bootstrap replicates.

### 2.3. Motif and Gene Structure Analysis

Five conserved motifs were identified from the 30 GmWIP amino acid sequences using MEME Suite v5.1.0 (https://meme-suite.org/meme/tools/meme, accessed on 22 April 2026) [24]. Conserved motif composition and exon-intron organization were integrated with soybean genome annotation and visualized using TBtools v2 [25].

### 2.4. Chromosomal Localization and Gene Duplication Analysis

Chromosomal positions of all 30 *GmWIP* genes were extracted from the soybean genome annotation. Chromosomal distribution and synteny were visualized using TBtools [25]. Duplication events were analyzed using MCScanX. Segmental duplications were defined as syntenic blocks across chromosomes, whereas tandem duplications were defined as two or more adjacent paralogous genes within a 200 kb region on the same chromosome with no intervening non-homologous genes.

### 2.5. Cis-Acting Element Analysis

Sequences 2000 bp upstream of each *GmWIP* gene were retrieved using TBtools [25]. Cis-acting elements were predicted using PlantCARE (https://bioinformatics.psb.ugent.be/webtools/plantcare/html/, accessed on 22 April 2026) [26].

### 2.6. Ka/Ks Calculation

For segmental duplicate pairs, coding sequences (CDS) were aligned based on their corresponding Clustal Omega protein alignments using a codon-guided custom script, with terminal stop codons removed. The non-synonymous (Ka) and synonymous (Ks) substitution rates were calculated using the Nei-Gojobori (NG86) method implemented in Biopython.

### 2.7. Tissue-Specific Expression Profiling of GmWIP Genes

Expression data for all 30 *GmWIP* genes across 11 soybean tissues (cotyledons, hypocotyls, shoot apices, primary leaves, trifoliate leaves, flowers, pods with seeds, seeds, axillary buds, and roots) were retrieved from the Soybean Expression Atlas. Expression values were ${log}_{2}(\mathrm{FPKM}+1)$ transformed and used for hierarchical clustering (using Euclidean distance and Ward’s minimum variance linkage method) and heatmap construction.

### 2.8. Plant Material and Salt Stress Treatment

Salt-tolerant cultivar Heinong 531 (HN531) and salt-sensitive cultivar Heinong 563 (HN563) were used as experimental materials. Seedlings were grown under standard greenhouse conditions (28 °C/22 °C day/night temperatures, 16 h light/8 h dark photoperiod, and 65% relative humidity). At the three-leaf stage, uniform seedlings were subjected to salt stress via root drenching with a 150 mM NaCl solution (applied gradually over 24 h to prevent osmotic shock and maintained for 48 h). Control plants were maintained under identical non-saline conditions. Root, stem, and leaf samples were collected simultaneously at 10:00 AM from three biological replicates per treatment.

### 2.9. RNA Extraction and RT-qPCR

Total RNA was extracted from roots, stems, and leaves using the FastPure Universal Plant Total RNA Extraction Kit (Vazyme Biotech, Nanjing, China). First-strand cDNA was synthesized from 1 µg total RNA using the HiScript III First Strand cDNA Synthesis Kit. RT-qPCR was performed using ChamQ SYBR qPCR Master Mix according to the manufacturer’s instructions. Relative gene expression was calculated using the 2^−ΔΔCt^ method, with *GmActin* (*Glyma.18G290800*) as the internal reference gene. Primer sequences are provided in Table S1. Primer amplification efficiencies (95–105%) and dissociation curves were verified prior to analysis. Three independent biological replicates, each with three technical replicates, were used for each genotype-tissue-treatment combination.

### 2.10. Physiological Assays

Malondialdehyde (MDA) content, an indicator of lipid peroxidation, was determined by the thiobarbituric acid (TBA) method [27]. Briefly, fresh leaf tissue (~0.5 g) was homogenized in 5% (*w*/*v*) trichloroacetic acid (TCA) and centrifuged at 12,000× *g* for 10 min. An aliquot of the supernatant was mixed with an equal volume of 0.6% (*w*/*v*) TBA prepared in 10% TCA, heated at 100 °C for 15 min, and then rapidly cooled on ice to stop the reaction. Absorbance was recorded at 532 nm, with non-specific turbidity corrected by subtracting the absorbance at 600 nm. MDA content was calculated using an extinction coefficient of 155 mM^−1^ cm^−1^ and expressed as nmol g^−1^ FW.

Proline content was determined using the sulfosalicylic acid extraction and acid-ninhydrin colorimetric method [28]. Leaf samples (~0.5 g) were homogenized in 3% (*w*/*v*) sulfosalicylic acid and centrifuged, after which the supernatant was reacted with equal volumes of glacial acetic acid and acid-ninhydrin reagent, heated at 100 °C for 1 h, and the reaction terminated on ice. Chromophores were extracted with toluene, and absorbance was measured at 520 nm against a toluene blank. Proline concentration was calculated from a standard curve (0–50 µg mL^−1^ L-proline) and expressed as µg g^−1^ FW.

Soluble sugar content was measured using the anthrone-sulfuric acid method [29]. Extracts were prepared by boiling leaf tissue in distilled water, and an aliquot of the extract was mixed with freshly prepared anthrone reagent (0.2% anthrone in concentrated H_2_SO_4_), heated at 100 °C for 10 min, and cooled before measuring absorbance at 620 nm. Glucose was used as the standard (0–100 µg mL^−1^), and results were expressed as mg g^−1^ FW.

Soluble protein content was determined by the Coomassie Brilliant Blue G-250 (Bradford) method [29]. An aliquot of crude enzyme extract was mixed with Bradford reagent, incubated at room temperature for 5 min, and absorbance was read at 595 nm. Bovine serum albumin (BSA) was used to generate the standard curve (0–1 mg mL^−1^), and protein content was expressed as mg g^−1^ FW.

Peroxidase (POD) activity was assayed by the guaiacol method. The reaction mixture contained 50 mM phosphate buffer (pH 7.0), 0.1 mM EDTA, 20 mM guaiacol, 10 mM H_2_O_2_, and enzyme extract. The increase in absorbance at 470 nm, corresponding to guaiacol oxidation, was monitored for 3 min at 30-s intervals, and activity was expressed as ΔA470 min^−1^ g^−1^ FW (or converted to U mg^−1^ protein using an extinction coefficient of 26.6 mM^−1^ cm^−1^, if normalized to protein content).

Catalase (CAT) activity was determined by monitoring the decomposition of H_2_O_2_ at 240 nm [30]. The reaction mixture consisted of 50 mM phosphate buffer (pH 7.0), 10 mM H_2_O_2_, and enzyme extract, and the absorbance decline was recorded over 3 min at 30-s intervals. Activity was calculated using the extinction coefficient of H_2_O_2_ (39.4 mM^−1^ cm^−1^) and expressed as µmol H_2_O_2_ decomposed min^−1^ g^−1^ FW (or mg^−1^ protein).

For all enzymatic assays, crude extracts were prepared by homogenizing fresh tissue (~0.5 g) in ice-cold 50 mM phosphate buffer (pH 7.0) containing 1% (*w*/*v*) polyvinylpolypyrrolidone (PVPP), followed by centrifugation at 12,000× g for 15–20 min at 4 °C; the resulting supernatant served as the crude enzyme source for POD and CAT determinations. All assays were performed with three biological replicates, each comprising three technical replicates.

### 2.11. Statistical Analysis

Data were evaluated using a two-way and three-way factorial ANOVA in SPSS v26.0 to test main effects and interaction terms. Post-hoc multiple comparisons were performed using Tukey’s honestly significant difference (HSD) test at p<0.05.

## 3. Results

### 3.1. Identification and Annotation of GmWIP Genes

A genome-wide search using the diagnostic C2H2 zinc finger domain (Pfam: PF00096) against the *G.max* Wm82.gnm4.ann1 reference genome identified a total of 30 putative *GmWIP* genes. These genes were designated *GmWIP1* through *GmWIP30* based on their physical order on the chromosomes (Table S2). Physicochemical analysis revealed that GmWIP proteins range from 237 to 521 amino acids in length, with molecular weights between 26.90 and 57.52 kDa. The theoretical isoelectric points (pI) varied from 5.76 to 9.40, reflecting a diversity of charge properties among family members. Subcellular localization predictions using WoLF PSORT and CELLO v2.5 consistently indicated that all 30 GmWIP proteins are localized to the nucleus, consistent with their putative roles as transcription factors.

### 3.2. Chromosomal Distribution and Gene Density

**Figure 1:**
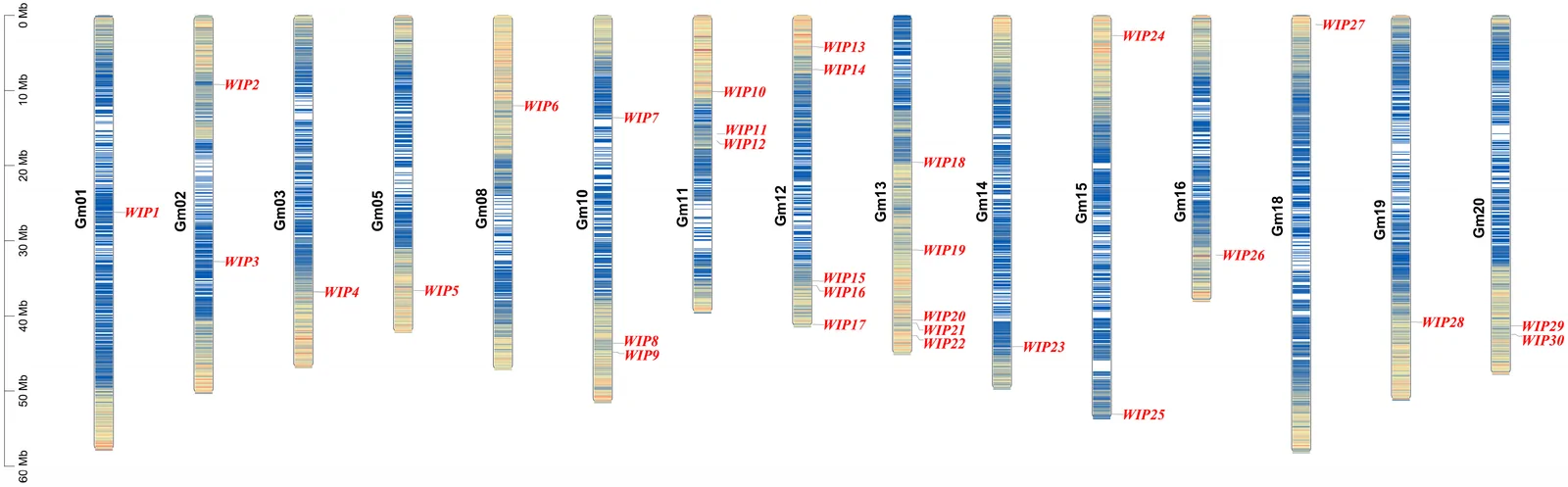
Chromosomal distribution of *GmWIP* genes in soybean. Physical distribution of the 30 *GmWIP* genes across the 20 soybean chromosomes (Gm01–Gm20). Chromosome ideograms indicate chromosome identity and relative physical length, and gene positions are shown according to their genomic coordinates. Chromosomes without identified *GmWIP* genes are retained for reference.

The 30 *GmWIP* genes are distributed across 15 of the 20 soybean chromosomes, showing a non-uniform density (Figure 1). Chromosomes 11, 12, and 13 were identified as “hotspots,” containing 5, 6, and 5 genes, respectively, which together account for 53.3% of the total family members. In contrast, chromosomes 1, 3, 5, 8, 10, 14, 15, 18, 19, and 20 each harbor only a single *GmWIP* locus. No genes were mapped to chromosomes 4, 6, 7, 9, or 17. The high density on chromosomes 11–13 suggests that these regions may have undergone more frequent segmental duplication or localized expansion during the paleopolyploidization events of the soybean genome.

### 3.3. Phylogenetic Analysis and Gene Structure

**Figure 2:**
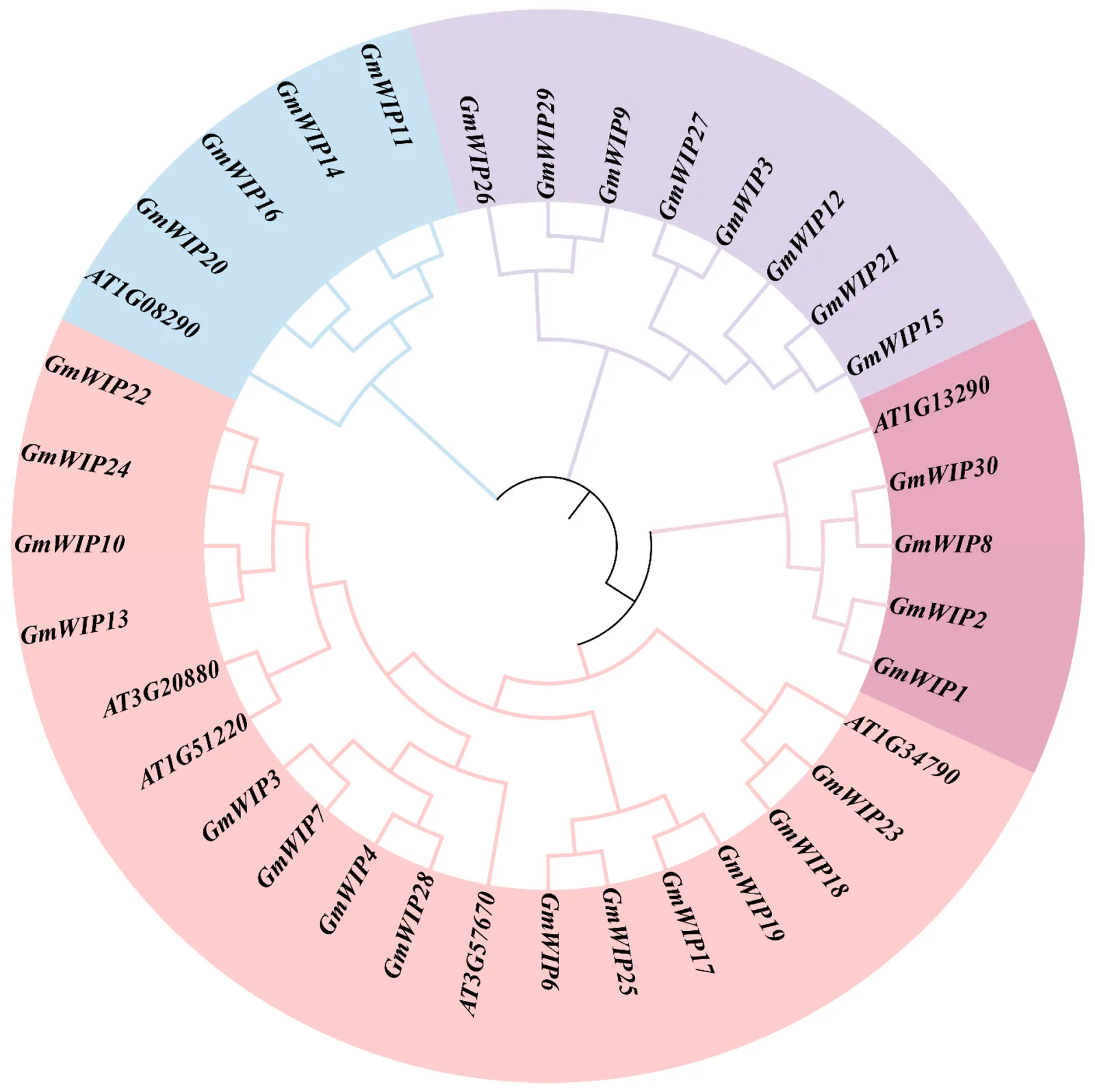
Phylogenetic relationships among GmWIP proteins from soybean and Arabidopsis. Maximum-likelihood phylogenetic tree constructed using 30 soybean GmWIP protein sequences and Arabidopsis WIP-family proteins. The tree was generated using the JTT+G model with 1000 bootstrap replicates. Bootstrap support values are indicated at the corresponding nodes. The four major phylogenetic groups are indicated by different colored sectors. Soybean GmWIP proteins are labeled GmWIP1–GmWIP30, and Arabidopsis proteins are labeled using their AGI identifiers.

Phylogenetic analysis based on 30 GmWIP proteins and Arabidopsis WIP homologs resolved the proteins into four major clades (Groups I–IV) (Figure 2). Groups I and II contained 8 and 10 soybean *GmWIP* members, respectively, whereas Groups III and IV each contained 6 soybean members. Several soybean genes occurred as closely related sister pairs or small clusters within individual clades. These relationships were supported by bootstrap values above 90% for several major nodes.

### 3.4. Gene Structure and Conserved Motif Analysis

**Figure 3:**
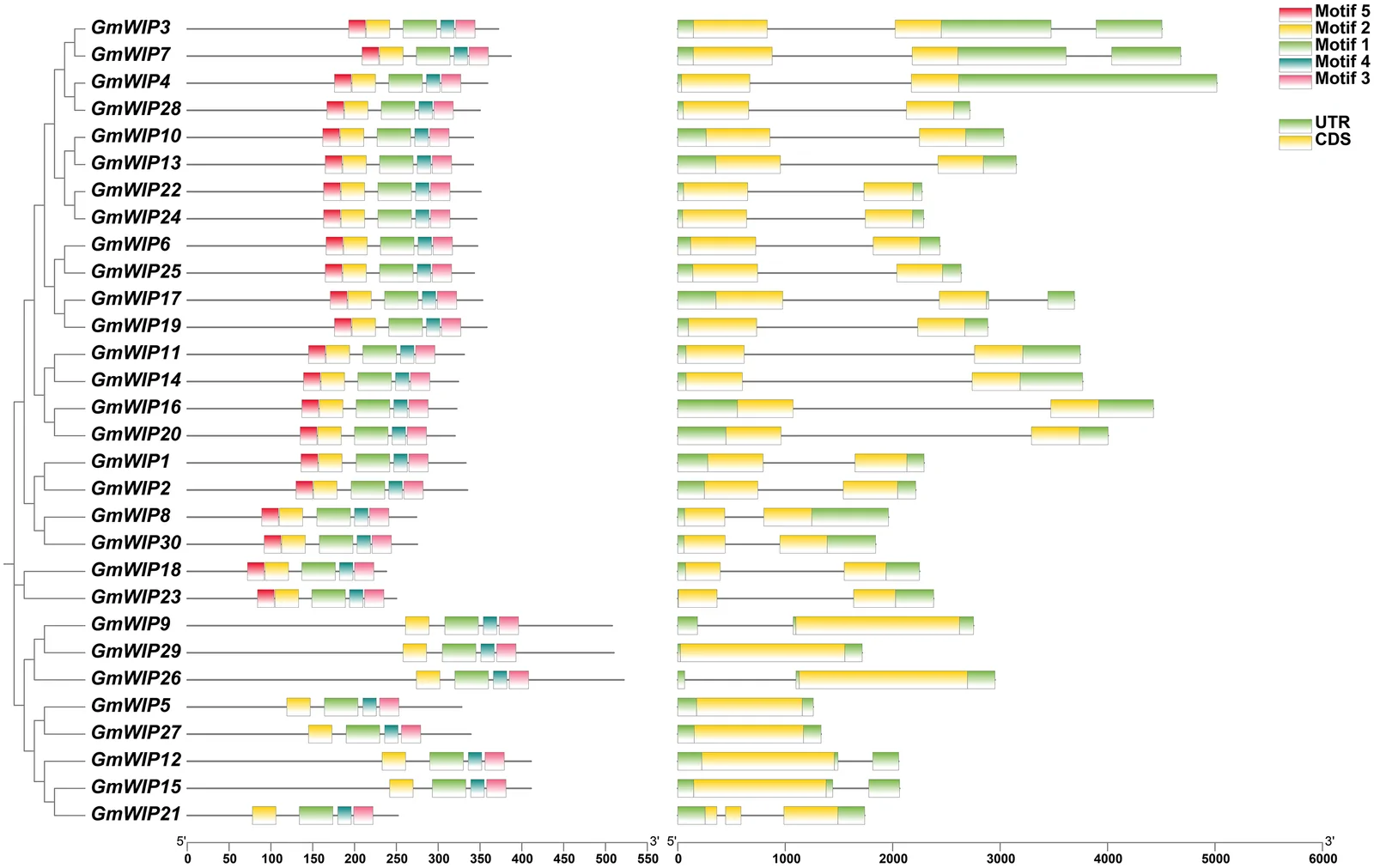
Conserved motif composition and gene structure of *GmWIP* family members. Conserved motif composition and exon–intron organization of the 30 GmWIP genes. Left: distribution of five conserved protein motifs identified using MEME. Right: exon–intron structures of the corresponding GmWIP genes. Genes are arranged according to their phylogenetic relationships. Motifs are numbered Motif 1–Motif 5, while UTRs and CDS regions are indicated separately.

Analysis of conserved protein motifs and exon–intron organization indicated substantial structural conservation among *GmWIP* genes within the same phylogenetic groups (Figure 3). Five conserved protein motifs (Motifs 1–5) were identified using MEME. Closely related GmWIP proteins generally exhibited similar motif composition and order, whereas proteins belonging to different phylogenetic groups showed greater variation. Most *GmWIP* genes contained two to four exons, with broadly similar exon–intron organization among closely related members. These structural patterns provide additional support for the phylogenetic classification of the *GmWIP* family.

### 3.5. Collinearity Analysis of GmWIP Genes

**Figure 4:**
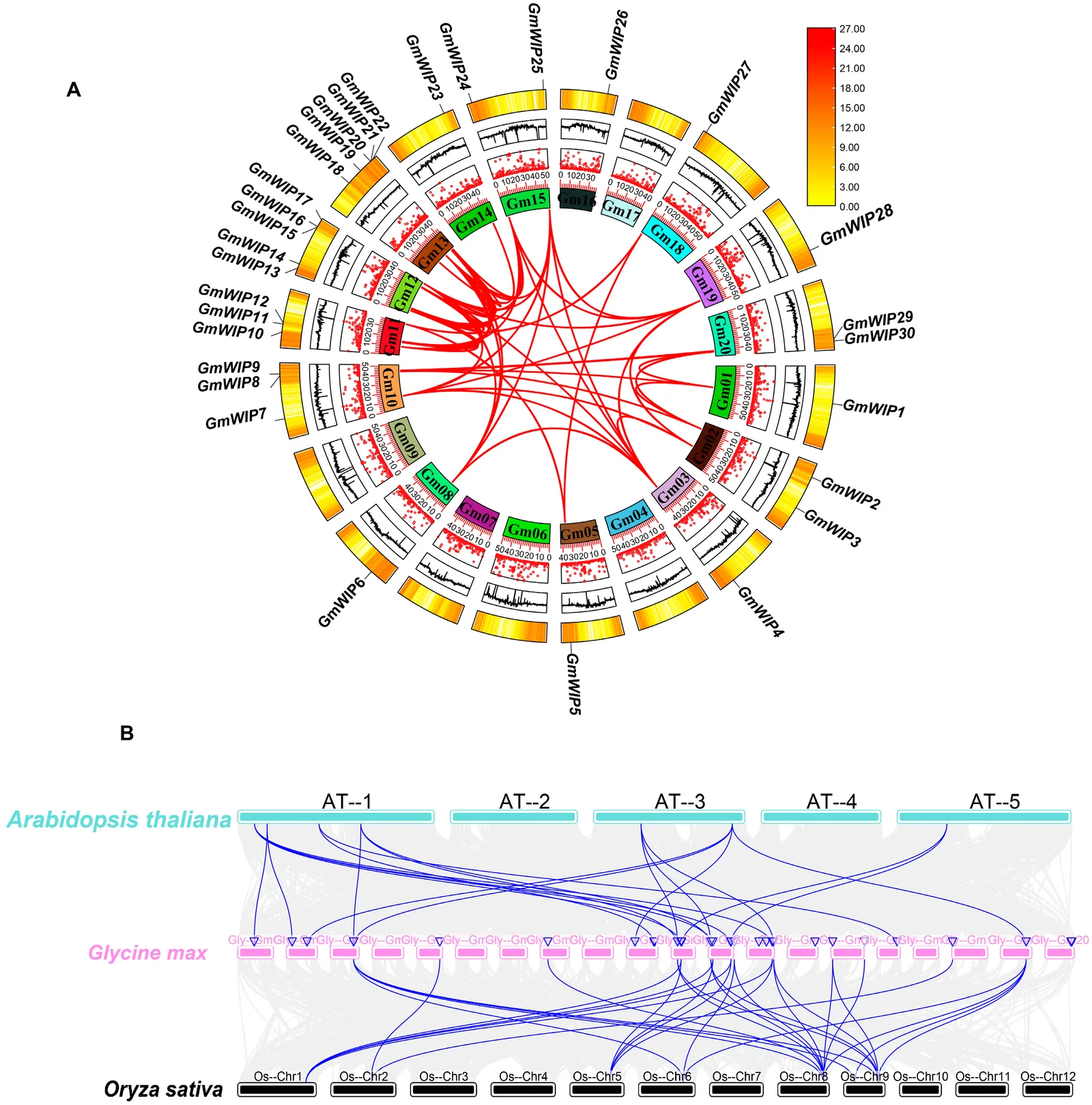
Collinearity analysis of *GmWIP* genes. (**A**) Intragenomic collinearity among *GmWIP* paralogs across the soybean genome. Outer tracks show, from outside to inside: chromosome ideograms color-coded by chromosome identity, gene/SNP density heatmaps, and a per-gene distribution track; red lines in the center connect syntenic *GmWIP* gene pairs. Chromosomes lacking *GmWIP* genes are represented with generic labels (Gm16–Gm20 region as applicable). (**B**) Interspecies macrosynteny among *A.thaliana*, *G.max*, and *O.sativa*. Blue lines connect orthologous gene pairs between species; grey lines in the background represent all detected syntenic blocks genome-wide, shown for context.

Intragenomic synteny analysis identified multiple segmental duplication events linking paralogous *GmWIP* gene pairs across different soybean chromosomes (Figure 4A), suggesting that whole-genome or segmental duplication has contributed substantially to the expansion of the *GmWIP* family in soybean. Interspecies macrosynteny analysis comparing soybean, *Arabidopsis*, and rice (Figure 4B) identified more collinear blocks between soybean and *Arabidopsis* than between soybean and rice, consistent with the closer evolutionary relationship between the two dicot species relative to the monocot outgroup. Ka/Ks (dN/dS) values were calculated for the 58 segmental duplicate *GmWIP* gene pairs identified above to assess the selection pressure acting on these paralogs since duplication. Ka/Ks could be computed for 54 of the 58 pairs; the remaining four pairs (*Glyma.05G177700–Glyma.11G240600*, *Glyma.11G192400–Glyma.12G081700*, *Glyma.12G081700–Glyma.12G179000*, and *Glyma.11G240600–Glyma.18G016700*) returned non-computable values, likely reflecting synonymous-site saturation between more divergent paralogs (Table S3). Among the 54 pairs with computable values, Ka/Ks ranged from 0.0351 to 0.4471 (mean ≈ 0.24), and all pairs fell below 1. The most strongly conserved pair, *Glyma.13G090000–Glyma.14G174900* (Ka/Ks = 0.0351, Ka = 0.0054), showed minimal nonsynonymous divergence, whereas the least constrained pair, *Glyma.08G154500–Glyma.13G203700* (Ka/Ks = 0.4471), still fell well within the range consistent with purifying selection. The majority of pairs (44 of 54) had Ka/Ks values between 0.1 and 0.3, with a smaller subset (9 pairs) exceeding 0.3 and only one pair fell below 0.1. This consistently low Ka/Ks distribution is consistent with purifying selection having acted broadly across the *GmWIP* gene family since duplication, constraining amino acid sequence divergence and suggesting that the functional core of these proteins has been preserved despite family expansion.

### 3.6. Cis-Acting Element Analysis of GmWIP Promoters

**Figure 5:**
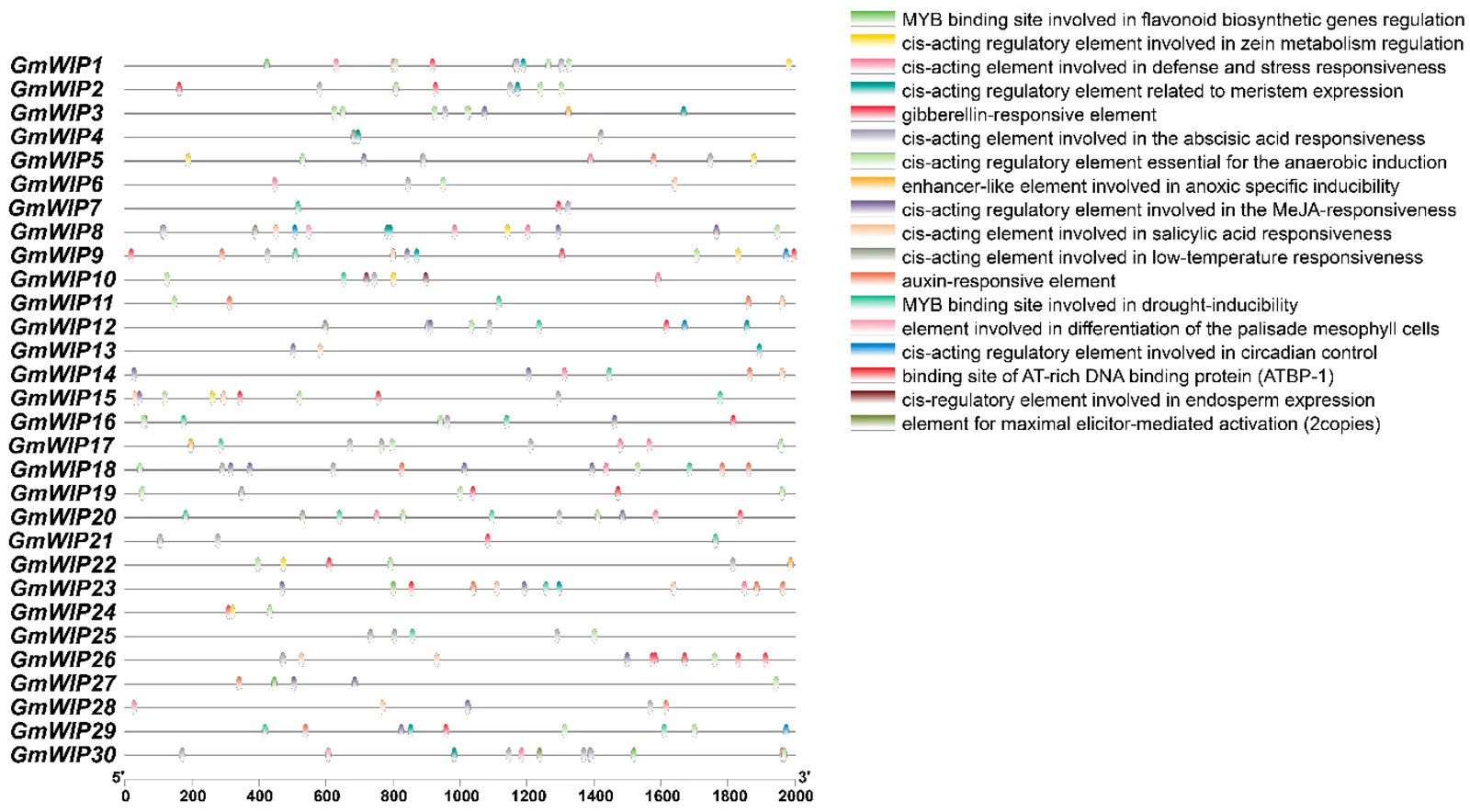
Cis-acting elements were predicted in 2000 bp upstream promoter sequences of all 30 *GmWIP* genes using PlantCARE and summarized via hierarchical clustering.

Cis-acting elements were predicted in 2000 bp upstream promoter sequences of all 30 *GmWIP* genes using PlantCARE and summarized via hierarchical clustering (Figure 5). *GmWIP* promoters are enriched for elements involved in phytohormone signaling, environmental stress responses, and growth and development. In the hormone response category, elements associated with abscisic acid (ABA), methyl jasmonate (MeJA), gibberellin (GA), salicylic acid (SA), and auxin were all identified. Certain members show pronounced enrichment for specific hormone-responsive elements: the promoters of WIP1 and WIP30 are notably ABA-responsive; WIP18 carries a high density of MeJA-responsive elements; and WIP26 is enriched for GA-responsive elements. Abiotic stress elements, including anaerobic induction elements, low-temperature-responsive elements, and drought-inducible MYB-binding sites, were found in most GmWIP promoters, suggesting broad stress-responsive regulatory potential. Elements linked to meristematic expression and regulation of zein metabolism were also identified in a subset of promoters.

### 3.7. Tissue Expression Analysis

**Figure 6:**
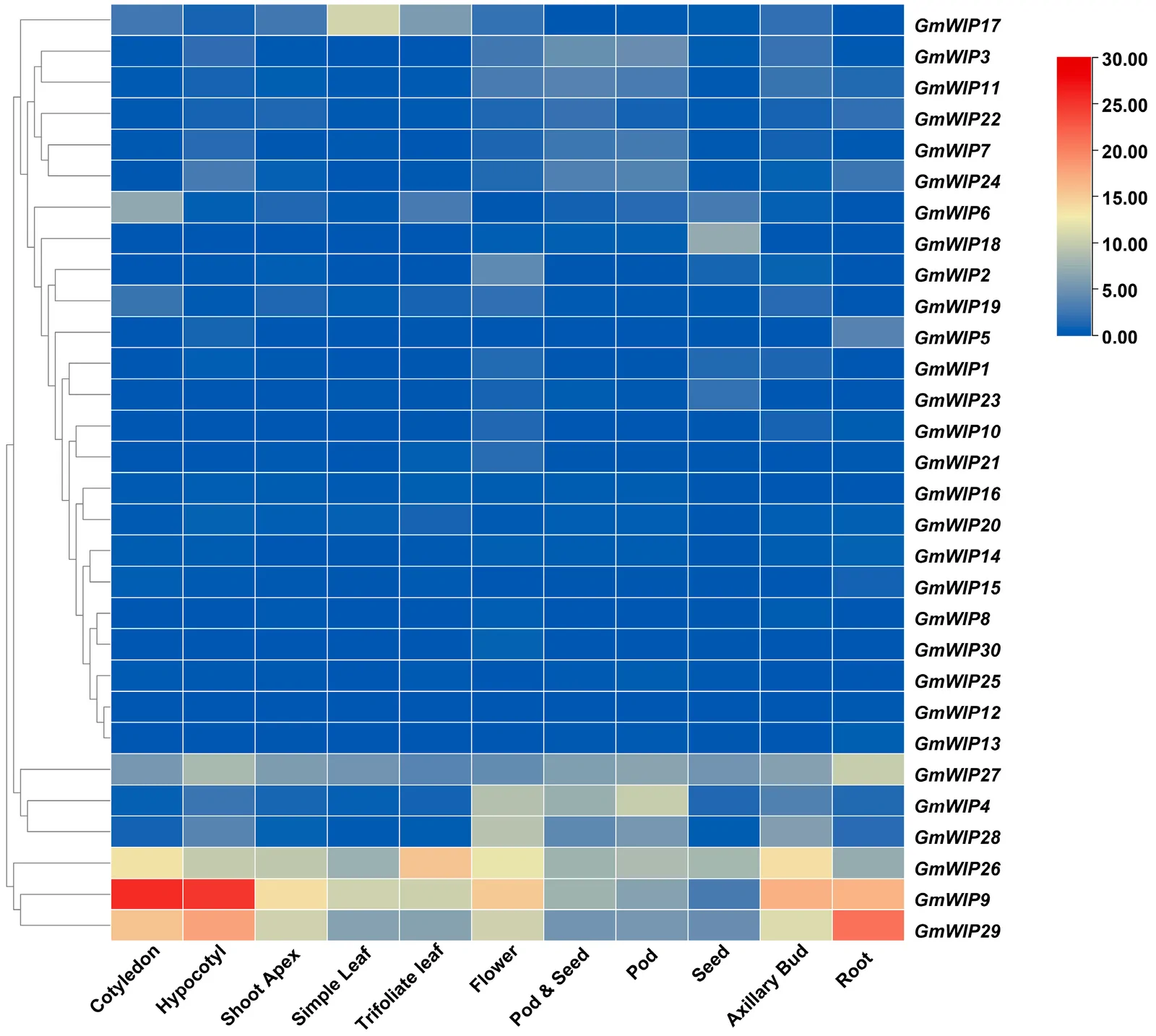
Tissue-specific expression profiles of *GmWIP* genes. Heatmap of *GmWIP* gene expression across eleven soybean tissues, with genes and tissues arranged by hierarchical clustering (dendrogram at left). Color scale indicates relative expression level (blue, low; red, high).

Expression profiling of the *GmWIP* gene family across eleven soybean tissues (cotyledon, hypocotyl, shoot apex, simple leaf, trifoliate leaf, flower, pod and seed, pod, seed, axillary bud, and root) revealed that the majority of family members were expressed at low and relatively uniform levels across all tissues examined. A small subset of genes, however, showed markedly elevated and tissue-preferential expression: *GmWIP9* and *GmWIP29* were most strongly expressed in cotyledon, hypocotyl, and root tissue, suggesting a possible role during early seedling establishment, while *GmWIP26* showed broader, moderately elevated expression across multiple tissues. *GmWIP4* and *GmWIP28* showed modest tissue-preferential elevation in reproductive tissues (flower and pod/seed-associated samples). Hierarchical clustering of expression profiles grouped these tissue-preferential genes separately from the broadly low-expressed majority of the family (Figure 6).

### 3.8. RT-qPCR Expression Analysis Under Salt Stress

**Figure 7:**
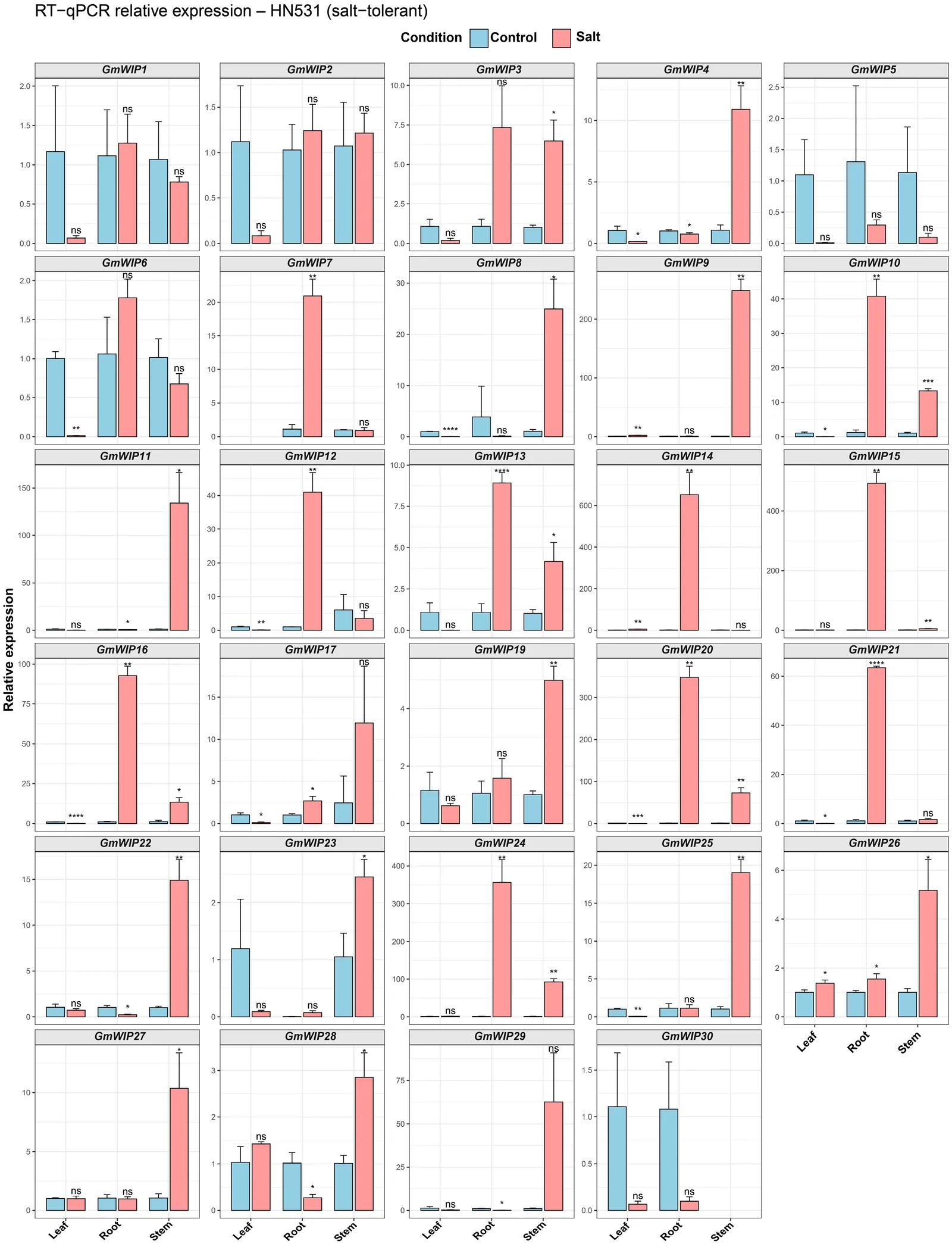
Salt-responsive expression of *GmWIP* genes in the salt-tolerant soybean cultivar HN531. Relative transcript abundance of *GmWIP* genes in leaves, roots, and stems of HN531 under control and salt-stress conditions. Relative expression levels were calculated using the 2^−ΔΔCt^ method with *GmActin* as the reference gene. Bars represent means ± SD of three independent biological replicates (n = 3). Statistical differences between control and salt treatments within each tissue were evaluated using Student’s *t*-test. * *p* < 0.05, ** *p* < 0.01, *** *p* < 0.001, **** *p* < 0.0001; ns, not significant.

To investigate the transcriptional responses of the *GmWIP* gene family to salt stress, the relative expression levels of *GmWIP* genes were analyzed by RT-qPCR in leaves, roots, and stems of the salt-tolerant soybean cultivar HN531 under control and salt-stress conditions (Figure 7). The *GmWIP* genes exhibited distinct tissue- and salt-responsive expression patterns. Several genes showed pronounced transcriptional induction following salt treatment, particularly in roots and stems. Among these, *GmWIP7*, *GmWIP9*, *GmWIP11*, *GmWIP12*, *GmWIP14*, *GmWIP15*, *GmWIP16*, *GmWIP20*, *GmWIP21*, *GmWIP24*, and *GmWIP29* displayed substantial increases in transcript abundance in at least one tissue under salt stress.

Other *GmWIP* genes showed more moderate responses or no significant changes in particular tissues. For example, *GmWIP1*, *GmWIP2*, *GmWIP5*, *GmWIP6*, *GmWIP13*, *GmWIP19*, *GmWIP23*, *GmWIP27*, and *GmWIP30* showed limited or tissue-dependent changes following salt treatment. Overall, the results indicate considerable variation in the magnitude and tissue specificity of *GmWIP* transcriptional responses to salinity in HN531.

**Figure 8:**
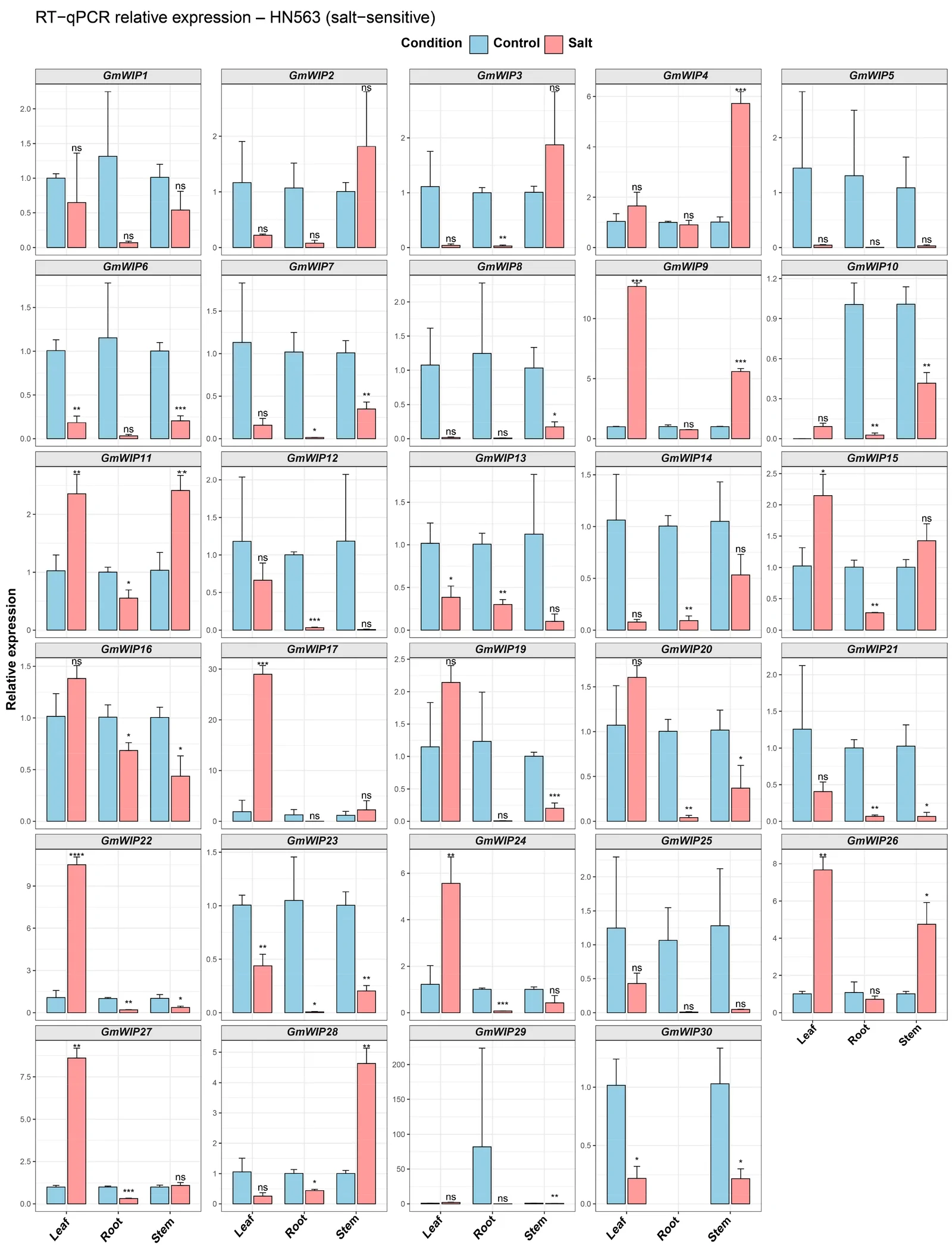
RT-qPCR expression of *GmWIP* genes in the salt-sensitive cultivar HN563 under salt stress. Relative transcript abundance of *GmWIP* genes in leaves, roots, and stems of HN563 under control and salt-stress conditions. Relative expression levels were calculated using the 2^−ΔΔCt^ method with *GmActin* as the reference gene. Bars represent means ± SD of three independent biological replicates (n = 3). Statistical differences between control and salt treatments within each tissue were evaluated using Student’s *t*-test. * *p* < 0.05, ** *p* < 0.01, *** *p* < 0.001, **** *p* < 0.0001; ns, not significant.

To further characterize the transcriptional response of the *GmWIP* gene family to salinity, the relative expression levels of *GmWIP* genes were examined in leaves, roots, and stems of the salt-sensitive soybean cultivar HN563 under control and salt-stress conditions (Figure 8). *GmWIP* genes exhibited distinct tissue-dependent responses to salt treatment, with several genes showing significant changes in transcript abundance in one or more tissues. The magnitude and direction of these responses varied among *GmWIP* genes and tissues, indicating heterogeneous transcriptional responses to salinity in HN563.

Several *GmWIP* genes showed pronounced transcriptional induction under salt stress, whereas others exhibited relatively weak responses or no significant changes in specific tissues. These differential expression patterns indicate that individual *GmWIP* genes respond differently to salinity and provide a set of candidate genes for subsequent functional analyses. However, the observed transcriptional changes represent associations with salt treatment and do not, by themselves, establish a causal role for individual *GmWIP* genes in salt tolerance or in the regulation of physiological traits.

### 3.9. Phenotypic and Physiological Characterization Under Salt Stress

**Figure 9:**
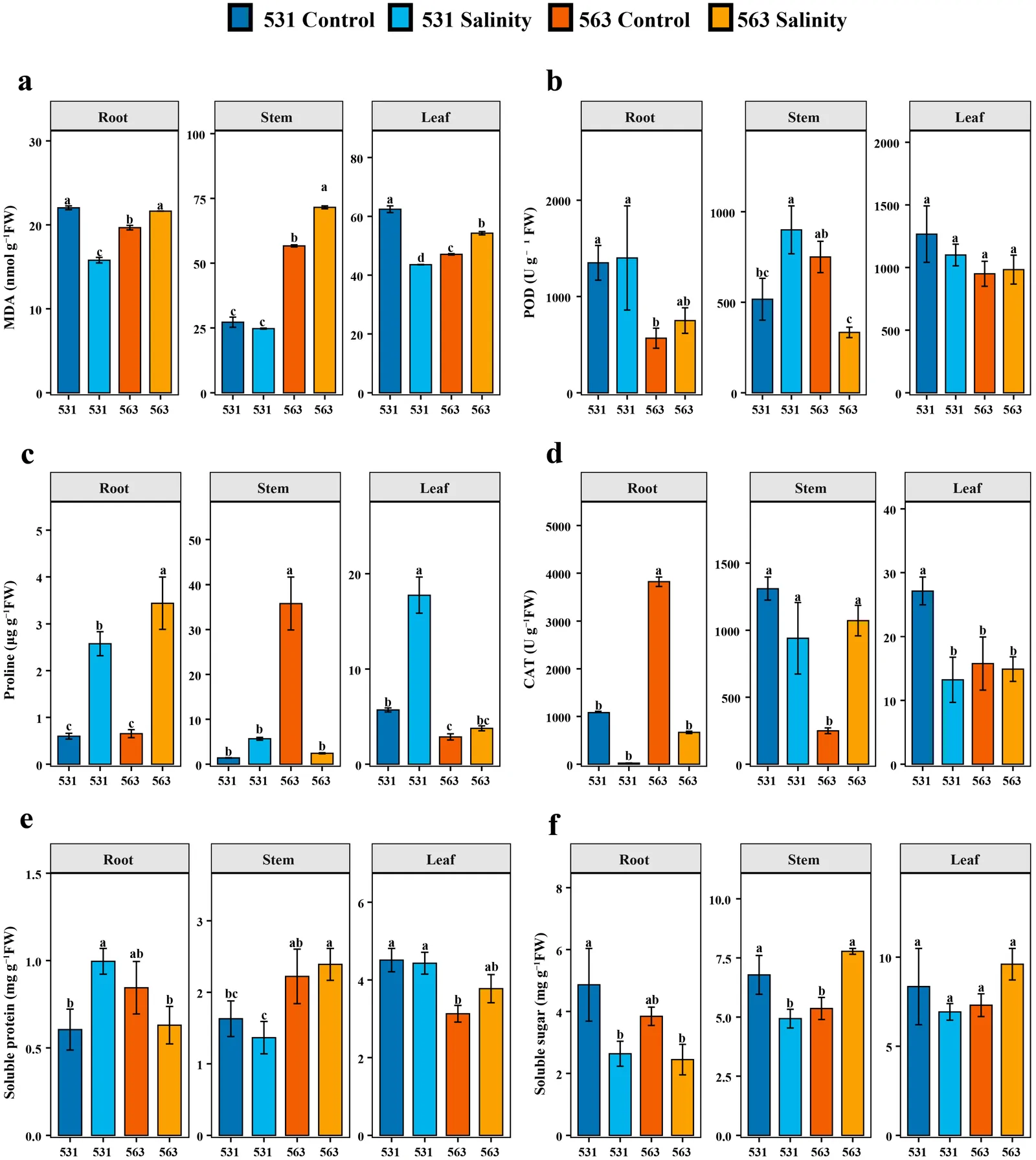
Physiological responses of soybean cultivars HN531 and HN563 to salinity stress. (**a**) Malondialdehyde (MDA), (**b**) peroxidase (POD) activity, (**c**) proline, (**d**) catalase (CAT) activity, (**e**) soluble protein, and (**f**) soluble sugar contents in roots, stems, and leaves of HN531 and HN563 under control and salinity conditions. Values are means ± SD of three independent biological replicates (n = 3). Data were analyzed using factorial ANOVA with genotype and salinity as fixed factors, followed by Tukey’s HSD test at *p* < 0.05. Different lowercase letters indicate significant differences among treatment combinations.

To assess the effects of salt stress on plant performance, whole-plant phenotypes and root morphologies were compared between HN531 and HN563 under control and salt-treated conditions. Under control conditions, both genotypes exhibited normal growth, with green, expanded leaves, sturdy stems, and well-developed root systems with abundant lateral roots. Under salt stress, the two genotypes responded markedly differently. HN563 showed severe symptoms: shoot growth was strongly inhibited and plants were visibly stunted; leaves exhibited wilting, chlorosis, and senescence. Root examination after washing revealed a near-complete loss of root biomass, with greatly shortened primary roots, sharply reduced lateral root number, and darkened coloration. HN531, by contrast, maintained relatively stable growth. While occasional mild yellowing or marginal necrosis was observed on older leaves, the general plant stature remained upright and upper leaves retained turgor and green coloration. Root systems remained largely intact after washing (Figure 9).

Malondialdehyde (MDA) is a product of membrane lipid peroxidation and serves as an indicator of oxidative damage under stress. Under control conditions, MDA content in HN531 leaves was approximately 62.2 μmol/g, higher than in roots (21.9 μmol/g) or stems (27.5 μmol/g). Following salt treatment, MDA levels in HN531 declined in all three tissues, reaching approximately 15.7 μmol/g in roots, 24.9 μmol/g in stems, and 43.5 μmol/g in leaves. In HN563, control MDA values were approximately 19.9 μmol/g (roots), 57.0 μmol/g (stems), and 47.3 μmol/g (leaves). Salt stress increased MDA in HN563 roots (21.6 μmol/g), stems (71.0 μmol/g), and leaves (53.9 μmol/g), indicating substantially more serious oxidative damage compared to HN531 under the same conditions. Under control conditions, proline (PRO) content in HN531 was relatively low across all tissues: approximately 0.60 μg/g in roots, 1.39 μg/g in stems, and 5.70 μg/g in leaves. Salt stress triggered significant PRO accumulation in HN531, with the most pronounced increase in leaves (from 5.70 to 17.76 μg/g), accompanied by increases in stems (from 1.39 to 2.58 μg/g). In HN563, control stem PRO content was unusually high (~35.78 μg/g) but fell sharply to 2.42 μg/g following salt treatment. Root and leaf PRO levels in HN563 showed minimal changes. This depletion of the stem proline pool in HN563 under salt stress suggests disruption of amino acid metabolism in the sensitive genotype.

Soluble sugar content in HN531 was broadly maintained under salt stress, with no statistically significant changes in roots, stems, or leaves (all *p* > 0.05). In HN563, salt stress caused a significant decline in root soluble sugar (from 3.84 to 2.45 mg/g; *p* < 0.05), while stem and leaf soluble sugar increased markedly (stems from ~5.36 to ~7.78 mg/g; leaves from 7.30 to 9.60 mg/g; *p* < 0.001). These contrasting patterns suggest that HN531 maintains carbon metabolic homeostasis under salt stress, whereas HN563 shows disrupted sugar partitioning. In HN531, salt stress did not significantly alter soluble protein content in roots, stems, or leaves (all *p* > 0.05), consistent with protein homeostasis in the tolerant genotype. In HN563, root and stem protein levels were not significantly affected, but leaf soluble protein increased significantly following salt treatment (from 3.13 to 3.78 mg/g; *p* < 0.05). This leaf-specific protein accumulation in HN563 may represent a passive osmotic adjustment response. In HN531, peroxidase (POD) activity was broadly maintained under salt stress across all tissues, with no statistically significant changes (all *p* > 0.05). Control values were approximately 1350 U/g (roots), 517 U/g (stems), and 1267 U/g (leaves); salt-stressed values were approximately 1400 U/g, 900 U/g, and 1100 U/g, respectively. In HN563, root and leaf POD activities were not significantly altered by salt treatment. However, stem POD activity in HN563 declined significantly under salt stress (from 750 to ~333 U/g; *p* < 0.001), indicating substantial impairment of antioxidant capacity specifically in the stem of the sensitive genotype. Catalase (CAT) activity showed pronounced tissue-specific baseline differences, with substantially higher activity in roots and stems than in leaves in both genotypes. In HN531, root CAT activity was approximately 1020 U/g under control conditions and declined to near-undetectable levels following salt treatment, while stem CAT activity was largely maintained (1320 U/g under control vs. 940 U/g under salt) and leaf CAT activity decreased modestly (27 to 13 U/g). In HN563, root CAT activity was markedly higher than in HN531 under control conditions (3860 U/g) and fell sharply to 595 U/g under salt stress, whereas stem CAT activity increased following salt treatment (from 225 to 1080 U/g); leaf CAT activity changed little (16 to 15 U/g). The sharp decline in root CAT activity under salt stress in both genotypes, most pronounced in HN531, suggests that root catalase-mediated H_2_O_2_ scavenging is particularly sensitive to salt-induced oxidative stress, whereas the contrasting stem response maintained in HN531 but induced in HN563 may reflect distinct compensatory antioxidant strategies between the two genotypes.

## 4. Discussion

WIP transcription factors belong to the A1d subgroup of C2H2-type zinc finger proteins and have documented roles in organ initiation, tissue differentiation, and reproductive development [31,32,33]. The present study provides a genome-wide characterization of the WIP transcription factor gene family in soybean, with particular emphasis on its genomic organization and transcriptional responses to salt stress. We identified 30 *GmWIP* genes and characterized their duplication history, evolutionary relationships, conserved motifs, gene structures, promoter cis-acting elements, tissue expression patterns, and responses to salt stress. The identification of numerous segmental duplicate pairs and the generally low Ka/Ks values indicate that the family has undergone substantial expansion while remaining under predominantly purifying selection.

The evolutionary conservation of GmWIP proteins is consistent with the established developmental functions of WIP proteins in other plant species [33]. WIP proteins contain a characteristic zinc-finger domain comprising multiple zinc-finger motifs, and functional studies in Arabidopsis have demonstrated roles for individual WIP members in seed coat development, transmitting tract formation, and other developmental processes [31,32,33]. These established functions provide a biological context for the conservation observed among soybean *GmWIP* family members. However, the present evolutionary analyses alone do not demonstrate that all *GmWIP* genes retain equivalent functions in soybean, and functional diversification among individual family members remains possible.

The promoter analysis further showed that many *GmWIP* genes contain cis-acting elements associated with ABA, MeJA, and other hormone- and stress-responsive pathways. These features indicate that *GmWIP* genes may be responsive to hormonal and environmental signals, but the presence of cis-acting elements does not by itself demonstrate their activity or establish a direct role in salt-stress signaling. The RT-qPCR results provide additional evidence that several *GmWIP* genes respond to salt treatment in a genotype- and tissue-dependent manner. In the salt-tolerant cultivar HN531, a broader and, for several genes, stronger transcriptional response was observed than in HN563, although the magnitude and direction of expression changes varied substantially among tissues and family members.

The transcriptional responses occurred alongside differences in physiological traits associated with salt-stress adaptation. HN531 generally maintained more stable physiological responses, including proline, soluble sugar, and protein levels and antioxidant enzyme activities, whereas HN563 exhibited more pronounced alterations in several of these traits. These observations indicate that differential *GmWIP* expression and physiological responses to salt stress occur in parallel and may identify candidate *GmWIP* genes associated with the salt-stress response. However, the present data do not establish that *GmWIP* genes regulate proline accumulation, osmotic adjustment, antioxidant activity, MDA accumulation, or other measured physiological traits. Thus, the observed relationships should be interpreted as associations rather than evidence of direct regulatory effects.

Recent studies in other crops provide useful context for this interpretation. In Brassica napus, genetic and molecular evidence has implicated *BnaWIP2* in salt-stress responses, including effects on germination and regulation of ABA-related genes [34,35,36,37].WIP-family genes have also been associated with saline-alkali responses in alfalfa and salt-responsive expression in tomato [36]. These studies support the possibility that WIP-family genes participate in stress-responsive regulatory networks; however, their mechanisms cannot be assumed to be conserved in soybean. In particular, the ABA-related mechanism reported for *BnaWIP2* represents a testable hypothesis rather than an established mechanism for *GmWIP* genes.

Taken together, the present results indicate that a subset of *GmWIP* genes is transcriptionally responsive to salt stress in a genotype- and tissue-dependent manner and that these responses occur alongside differences in physiological traits associated with salt tolerance. The identified genes therefore represent promising candidates for future functional investigation. Definitive determination of whether individual *GmWIP* genes contribute to salt tolerance, and whether they directly regulate osmotic adjustment, antioxidant defense, ABA signaling, or related pathways, will require genetic and molecular approaches such as overexpression, CRISPR/Cas-mediated loss-of-function, complementation, target-gene analysis, and/or protein–DNA interaction assays.

## 5. Conclusions

This study provides a genome-wide identification and characterization of the WIP transcription factor gene family in soybean, identifying 30 *GmWIP* genes and characterizing their chromosomal distribution, phylogenetic relationships, gene structure, duplication history, conserved motifs, and promoter cis-acting elements. Expression profiling and RT-qPCR analysis indicated that a subset of *GmWIP* genes respond differentially to salt stress in a tissue- and genotype-dependent manner, with the salt-tolerant cultivar HN531 showing a broader and more pronounced transcriptional response than the salt-sensitive cultivar HN563. Physiological comparisons further indicated that HN531 maintained more stable proline accumulation, soluble sugar and protein homeostasis, and antioxidant enzyme activity under salt stress relative to HN563, consistent with more effective osmotic and oxidative stress management in the tolerant genotype. Together, these findings suggest that specific *GmWIP* family members may contribute to the soybean salt-stress response and provide a set of candidate genes, together with the underlying genomic resource, for future functional validation and potential application in soybean salt-tolerance breeding.

## Data Availability

The datasets generated and analyzed during the current study are available from the corresponding author upon reasonable request.

## Supplementary Materials

The following supporting information can be downloaded at: https://www.mdpi.com/article/10.3390/genes17080968/s1, Table S1: Primer sequences used for RT-qPCR analysis; Table S2: List of the 30 identified *GmWIP* genes, including gene ID, chromosomal location, and physicochemical properties; Table S3: Ka/Ks (dN/dS) values for the 58 segmental duplicate *GmWIP* gene pairs.
